# Serological and molecular detection of spotted fever group *Rickettsia* in a group of pet dogs from Luanda, Angola

**DOI:** 10.1186/s13071-017-2216-3

**Published:** 2017-05-31

**Authors:** Patrícia F. Barradas, Hugo Vilhena, Ana Cristina Oliveira, Sara Granada, Irina Amorim, Paula Ferreira, Luís Cardoso, Fátima Gärtner, Rita de Sousa

**Affiliations:** 1 Institute of Biomedical Sciences Abel Salazar (ICBAS), University of Porto, Oporto, Portugal; 2 Animal and Veterinary Research Centre (CECAV), University of Trás-os-Montes e Alto Douro (UTAD), Vila Real, Portugal; 3 Department of Veterinary Medicine, Escola Universitária Vasco da Gama, Coimbra, Portugal; 4 Baixo Vouga Veterinary Hospital, Águeda, Portugal; 5 Casa dos Animais Veterinary Clinic, Luanda, Angola; 6 Instituto de Inovação e Investigação em Saúde (i3S), Universidade do Porto, Oporto, Portugal; 7 Institute of Molecular Pathology and Immunology of the University of Porto (IPATIMUP), Oporto, Portugal; 8 Instituto de Biologia Molecular e Celular (IBMC), Universidade do Porto, Oporto, Portugal; 9 Department of Veterinary Sciences, School of Agrarian and Veterinary Sciences, UTAD, Vila Real, Portugal; 10 National Institute of Health Dr. Ricardo Jorge (INSA), Lisbon, Portugal

**Keywords:** Angola, Dogs, *Rickettsia*, Seroprevalence, Spotted fever group

## Abstract

**Background:**

Infections with tick-borne rickettsiae can cause diseases well known in humans but still not so well characterized in dogs. Susceptibility to infection depends on the virulence of *Rickettsia* spp. and only a few of them have been described to cause disease in dogs. The aim of this study was to investigate the exposure to *Rickettsia* spp. among a group of pet dogs from Luanda, Angola.

**Results:**

Out of 103 dogs included in the study, 62 (60.2%) were infested with ticks. Plasma specimens tested for serology by an immunofluorescence assay (IFA) revealed that six (5.8%) dogs had detectable immunoglobulin G (IgG) antibodies to spotted fever group *Rickettsia* (SFGR), with endpoint titers of 64 for two dogs, 128 for three dogs and 1024 for one dog. From the seropositive group of dogs, five (83%) of them were males, with their age ranging from 1 to 8 years old. Among the seropositive dogs, four (66.7%) were parasitized with ticks and no breed (or cross) was found to be associated with specific antibodies. *Rickettsia* spp. DNA was detected by nested-polymerase chain reaction (PCR) in two (1.9%) dogs that were found to be seronegative.

**Conclusions:**

Seroprevalence and molecular detection of *Rickettsia* spp. infection in this group of pet dogs from Luanda is low compared with other studies performed in the same type of hosts in other areas. Although many dogs were parasitized with ticks, a low prevalence of *Rickettsia* spp. could be related with the hypothesis of a low rickettsial prevalence in the infesting ticks. This study provides evidence that dogs in Luanda are exposed to *Rickettsia* spp., but further studies are needed to better characterize the bacterial infections in dogs and in their ectoparasites.

## Background

Canine vector-borne diseases (CVBD) are currently a focus of increasing attention, due to their clinical importance in veterinary medicine and also in public health [1]. The agents that cause CVBD are transmitted by hematophagous arthropods such as ticks, fleas and mosquitoes [2]. Dogs are hosts for these ectoparasites, allowing them to feed on blood in order to complete their life-cycle. When infected, these ectoparasites may transmit the pathogens to dogs, which in turn may develop clinical manifestations or remain apparently healthy although infected, but potentially acting as a reservoir for those agents [3].

Tick-borne rickettsiae are well known to cause disease in humans, but knowledge on the susceptibility of dogs to different *Rickettsia* spp. and related illnesses is still limited. Dogs can develop transient rickettsiemia and show clinical signs; however, they are not considered reservoirs of *Rickettsia* spp. [4]. Since dogs are often exposed to infected ectoparasites, they develop antibodies against these agents and can be used as sentinels, i.e. to reveal the presence of pathogens that are circulating in a particular geographical region [5–7]. Nevertheless, due to cross-reactivity, serology does not allow the discrimination of *Rickettsia* spp. within the spotted fever group (SFGR) or typhus group rickettsiae (TGR). Under this circumstance, molecular detection and sequencing are the methods that enable *Rickettsia* spp. identification [6, 8, 9].

Up until now, only *Rickettsia rickettsii* [10, 11] and *Rickettsia conorii* [6, 12] have been described to cause diseases in dogs. In Africa, SFGR has been detected in ticks and human patients [13], but only a few studies have been carried out in dogs [14]. To better understand if rickettsiae are circulating in dogs in Luanda, this study aimed to evaluate the seroprevalence and to characterize *Rickettsia* spp., using molecular tools, in a convenience sample of dogs from Luanda.

## Methods

### Dogs

From January to February of 2013, a total of 103 pet dogs presented to a veterinary clinic in Luanda city, Angola were included in this study. Dogs were targeted by convenience sampling and comprised two groups: (i) apparently healthy animals that were brought in for prophylactic procedures, including vaccination or deworming; and (ii) dogs with a clinical suspicion of CVBD, presenting with at least one of the following clinical manifestations: anorexia, weight loss, fever, dehydration, onychogryphosis, lymphadenomegaly, gastrointestinal alterations, jaundice, dermatological or ocular abnormalities, anemia, thrombocytopenia, leukocytosis or leukopenia, hyperproteinemia or hyperglobulinemia.

For all the dogs included in the study, a questionnaire with epidemiological, clinical and laboratory data was completed. EDTA-anticoagulated whole blood specimens were collected from each dog. Blood specimens were centrifuged and plasma and buffy-coat were separated and stored at −20 °C.

### Serological analysis

IgG antibodies against *Rickettsia* spp. were detected by an in-house IFA using a *Rickettsia africae* strain as antigen, prepared at the Portuguese National Institute of Health, as previously described [15]. The cut-off for a positive result was considered at a titre of IgG ≥ 128, and a titer of 64 was considered suspected of contact, based on previous studies in Portuguese dogs [5].

### Molecular analysis

Total genomic DNA was extracted from 400 μl of peripheral mononuclear cells (buffy-coat) using a commercial kit (E.Z.N.A.® Blood DNA Mini Kit, Omega Bio-Tek, Norcross, GA, USA), according to the manufacturer’s instructions.

*Rickettsia* DNA in blood was screened by nested-polymerase chain reaction (PCR) targeting a fragment of the outer membrane protein B (*Omp*B) gene, as previously described by Choi et al. [16]. The first PCR reaction was performed using the set of primers R*omp*B OF and R*omp*B OR, which amplify a 511 bp fragment, and was followed by a second PCR reaction with internal set of primers of R*omp*B SFG-IF and R*omp*B SFG-IR, which amplify a 420 bp fragment [16]. For each reaction, water and *R. rickettsii* were included as negative and positive controls, respectively.

The PCR products of the expected size were purified with ExoSAP-IT PCR Product Cleanup (Affymetrix, Santa Clara, CA, USA) and were sequenced with the Big-Dye Terminator Cycle Sequencing kit (Applied Biosystems, Foster City, CA, USA) on an ABI 377 DNA sequencer according to the manufacturer’s recommendations. The sequencing reactions were performed with the forward and reverse primers used for PCR amplification. The sequences were analysed with Lasergene software v.7.0.

## Results

Out of the 103 tested dogs, 61 were males. Ages ranged from 3 to 168 months old (median: 12 months; inter-quartile range: 7.3–48). Eighty (77.7%) were outdoor-living dogs and 62 (60.2%) were infested with ticks. Based on physical and clinicopathological examination, 49 (47.6%) dogs were classified as apparently healthy and 54 (52.4%) dogs as clinically suspected of a CVBD.

SFGR-specific IgG antibodies were detected in six (5.8%) dogs, comprising two suspected dogs (i.e. with an endpoint titer of 64) and four positive dogs (three dogs with an endpoint of 128 and one dog with 1024).

From the seropositive group of dogs, five (83%) were males, with ages ranging from 1 to 8 years old, and all lived outdoors. Four (66.7%) of the six dogs with detectable antibodies were parasitized by ticks and no breed (or cross) was found to be associated with the presence of antibodies. Differential diagnostic showed that four (66.7%) of the dogs with antibodies to *Rickettsia* spp. were co-infected with single or multiple pathogens (molecular data not shown), such as *Anaplasma platys* [17], *Hepatozoon canis* [17], *Leishmania infantum* [18] or *Toxoplasma gondii* [19].

*Rickettsia* DNA was detected in two (1.9%) dogs, but sequence analysis was not able to identify species. One of these two dogs presented clinical manifestations compatible with CVBD, namely fever and anemia. None of those two dogs was seropositive for *Rickettsia* spp. by IFA. The clinically suspected dog was co-infected with *Babesia* spp. (molecular data not shown) [17].

## Discussion

To the best of our knowledge, this study shows for the first time the presence of SFGR in dogs in Luanda. A considerably lower prevalence of IgG antibodies against *Rickettsia* spp. was found in dogs when compared with other studies that assessed serum samples from dogs [11] or people [16]. On the other hand, the molecular prevalence in the present study was also lower than in another study that assessed *Rickettsia* spp. in dogs from Nigeria [12]. Nevertheless, the low prevalence could be related to the fact that tick species that were infesting this group of dogs had a low prevalence of rickettsial infection. For example, it has been described that *Amblyomma* spp. ticks, regarded as the vector of *R. africae*, are rarely found parasitizing dogs [20–23]. Other studies performed in Africa have revealed a high prevalence of *Rickettsia* spp. in dogs [24], as well as a high prevalence of pathogenic rickettsiae in ticks collected from domestic animals and cattle from Benin [22], Kenya [23] and Nigeria [25]. In North Africa the prevalence of infection in ticks matches that from southern Europe [24, 26–28], whereas in sub-Saharan Africa their prevalence is even higher, a situation which seems to be related with the distribution of *Amblyomma* spp. ticks [29]. One limitation of this study was the fact that it was not possible to identify the tick species that were infesting dogs. If they were infected with *Rickettsia* spp., additional data could help to explain the low prevalence of antibodies found in this group of dogs.

Seroepidemiological studies have used dogs and other canids as sentinels for the presence of *Rickettsia* spp. infection [5, 30]; however, and since serological cross-reactions within SFGR do not allow species differentiation, we have used molecular tools to characterize the *Rickettsia* spp. that could be circulating in the blood of dogs and could be associated with clinical illness [6, 9]. Nevertheless, in two dogs positive to *Rickettsia* DNA it was not possible to have a good quality of sequences to characterize *Rickettsia* spp. The positive PCR results in dogs without antibodies are concordant with what we found in humans [31]. In general, during an acute infection, when it is possible to detect rickettsiae DNA in blood, the host had not enough time to produce antibodies [32]. Most of the dogs are exposed during their lifetime to multiple pathogens to which they develop antibodies and it is very difficult to conclude about the canine rickettsial diseases based only in one testing sample. Testing two consecutive samples with 2-week intervals is advisable to detect seroconversion. Molecular detection for differential diagnosis is also very useful to distinguish CVBD agents. In this same group of dogs from Luanda, Cardoso et al. [17] by PCR identified five distinct tick-borne pathogens, *A. platys* (17.5%), *Ehrlichia canis* and *H. canis* (5.8%, each), *Babesia vogeli* (1.0%), *Babesia gibsoni* and an unnamed *Babesia* sp. (1.0%). Other two studies done with the same group of dogs detected the presence of *L. infantum* (1.9% by serology and 1.0% by PCR) [18] and of *T. gondii* (seroprevalence of 15.5%) [19].

New studies are needed to better characterize the circulation of *Rickettsia* spp. in vertebrate hosts and vectors in Angola, in order to try and understand their epidemiology and clinical importance in domestic and free-roaming dogs.

## Conclusions

To the best of our knowledge, this is the first study that provides evidence for the presence of *Rickettsia* spp. in a canine population from Luanda, Angola. Further investigations, including a larger population of domestic and free-roaming dogs from different urban and rural provinces of Angola and vectors of *Rickettsia* spp., are necessary to better understand the epidemiology and clinical importance of these vector-borne agents.

## Abbreviations

CVBD: Canine vector-borne diseases
IFA: Immunofluorescence assay
IgG: Immunoglobulin G
PCR: Polymerase chain reaction
SFGR: Spotted fever group *Rickettsia*
